# A Single Axial Slice of the Sternocleidomastoids and Paravertebral Muscles Associated with Worse Local Progression-Free Survival and Severe Toxicity in Sarcopenic Head and Neck Cancer Patients Undergoing Radiotherapy

**DOI:** 10.7759/cureus.22463

**Published:** 2022-02-21

**Authors:** William Jin, Benjamin Rich, Raphael Yechieli, Laura Freedman, Michael A Samuels, Matthew Abramowitz, Ruben Carmona, Stuart E Samuels

**Affiliations:** 1 Department of Radiation Oncology, Jackson Memorial Hospital, Miami, USA; 2 Department of Radiation Oncology, University of Miami, Miami, USA

**Keywords:** chemoradiation, radiotherapy, cachexia, head and neck cancer, sarcopenia

## Abstract

Objective

The objective of this study is to contrast the predictive ability of targeted muscle groups as radiographic proxies of sarcopenia on computerized tomography (CT) with body mass index (BMI) in head and neck cancer patients (H&NCP) undergoing radiation at a safety net hospital, and to evaluate sarcopenia with survival, local progression, toxicities and treatment delays.

Methods

A retrospective review included 52 H&NCP treated between 2017-2019. The posterior neck muscles (PN), sternocleidomastoids (SCM), and their summed volume (AM) were contoured at C3 on patients’ pre-treatment CT scans, then normalized to obtain skeletal muscle index (MI) values. Pre-treatment BMI was also evaluated. Cutoffs for sarcopenia were determined by receiver operating characteristic curves. Overall survival and local recurrence-free survival were evaluated by Kaplan-Meier. Acute grade 3 or higher toxicities were evaluated by binomial logistic regression.

Results

Using all neck muscles (AM-MI) produced the best model for predicting outcomes, outperforming individual muscle groups and BMI. Local progression-free survival was worse in sarcopenic patients at 25.81 months versus 35.40 months (p=0.026). Acute grade 3 or higher toxicities were associated with sarcopenia (p=0.005).

Conclusions

In this small, retrospective single-institution experience at a safety net hospital, a single axial slice of the combined sternocleidomastoids and paravertebral muscles at C3 performed better than either muscle group alone or pre-treatment BMI at predicting oncologic outcomes.

## Introduction

Head and neck cancer patients (H&NCP) must undergo one of the most difficult combined modality treatments for cancer. In a typical seven-week course of concurrent chemoradiation, up to 85% [1] of patients develop symptoms severe enough to need active, supportive interventions. The process of cancer cachexia relies on pro-inflammatory cytokines like TNF-α [2], IL-1, and IL-6 to create a hypercatabolic state capable of meeting the anabolic demands of a perpetually growing tumor [3-5]. Without interventions, this leads to sarcopenia, a clinical phenotype characterized by a quantifiable loss of skeletal muscle mass and function [6]. Recent studies have shown the impact of sarcopenia on both treatment tolerance and outcomes in H&NCP during chemoradiation [7-10]. The importance of this is understated, as sarcopenia is a potentially modifiable risk factor amenable to intervention.

Traditional metrics such as pre-treatment weight and body mass index (BMI) fail to reliably identify high-risk H&NCP. Radiographic proxies for sarcopenia were first identified in gastrointestinal cancers using a single axial slice of the bilateral psoas muscles [11]. The large hip flexors were selected as proxies for sarcopenia for two reasons. These muscles were conveniently available as part of the standard diagnostic workup for gastrointestinal cancers. In addition, the hypothesis that form begets function was confirmed as the psoas correlated with both a quantitative loss of muscle mass and function. In gastrointestinal cancers, emerging evidence suggests correlations with both survival and toxicity [12-19]; however, an equivalent proxy for H&NC has yet to be determined.

The ideal muscle group should be reproducibly identified on cross-sectional imaging and functions in some capacity as a tonic (or postural) muscle. In contrast to phasic muscles, tonic muscles are critical to postural function and resistant to disuse atrophy, with size correlating to function. While there is growing evidence that the psoas muscles are applicable to H&NCP undergoing chemoradiation [9,20], most of these patients do not undergo high-resolution CT scans of the abdomen/pelvis as part of their oncologic workup. Efforts were directed to find a localized proxy in diagnostic H&N scans [21,22]. A Pamukkale University study aimed to identify the ideal H&N correlate to the psoas muscles. They looked at the paravertebral muscles (C2, C3, C4) and the sternocleidomastoids, revealing the C3 paravertebral muscles correlated best for men and sternocleidomastoids for women [23]. However, these previous studies used a semi-quantitative thresholding technique requiring images to be transferred to an external software program for analysis.

Our study aimed to identify a clinically meaningful cutoff for sarcopenia in our own institutional cohort of H&NCP at a safety net hospital using clinically identified muscle groups and contrast the predictive value of the sternocleidomastoids (SCM) alone, the posterior neck (PN) muscles alone, and both with pre-treatment BMI. Finally, we aimed to evaluate the utility of the most optimal sarcopenia proxy with important endpoints, like treatment outcomes, tolerance, and delays. We hypothesized when identified, sarcopenia predicts worse local control, worse survival, increased acute toxicity, and increased delays in H&NCP undergoing radiotherapy.

## Materials and methods

Patient selection and treatment

A retrospective review of an IRB-approved head and neck cancer database at a safety net hospital was performed. Sixty-three patients that underwent definitive head and neck treatment with radiation at a safety net hospital and with at least twelve months of follow-up were included. Then, nine patients were excluded because of secondary or metachronous cancers, prior history of cervical trauma, prior history of cervical radiotherapy, cervical kyphosis, cervical scoliosis, significant cervical positional lordosis, or paravertebral muscle invasion.

All patients were treated with radiation delivered via intensity-modulated radiation therapy. Use of chemotherapy was decided in a multidisciplinary tumor board. Demographic and outcomes data were obtained from the electronic medical record; radiation treatment data were collected from the Eclipse treatment planning system (TPS).

Skeletal muscle delineation

**Figure 1:**
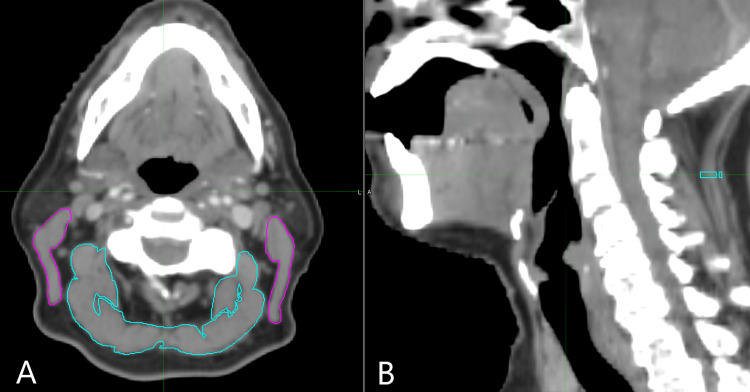
Delineation of muscle groups on axial and sagittal computerized tomography scans Pre-treatment contrast-enhanced computer tomography scans with (A) axial view at the C3 vertebral body and (B) midplane sagittal view. Magenta highlights sternocleidomastoids and cyan highlights posterior neck muscles.

H&N images used for radiation planning were transferred from the TPS to MiM version 6.7.6 64-bit edition (MIM Software Inc; Beachwood, OH). The first visible axial slice with the entire C3 vertebral body and the posterior process was chosen for contouring. Two muscle volumes were segmented: the PN and the bilateral SCM. A third volume (AM) was created from the sum of the PN and SCM. The PN included the longissimus capitis, levator scapulae, semispinalis capitis, and splenius capitus (Figure 1); transversospinales was excluded as it was visualized on some images entirely and trapezii were omitted as they are primarily phasic muscles.

Defining sarcopenia

Muscle Indices (MI) were produced by normalizing by height using the equation shown below [12].

Muscle index (mm^2^/m^2^) = muscle cross sectional area (mm^2^)/patient height (m^2^)

Four indices were generated from running receiver operating characteristic (ROC) curves with respect to crude two-year local progression on each of the three segmented volumes (posterior neck muscle index (PN-MI), sternocleidomastoid muscle index (SCM-MI), sternocleidomastoid and posterior neck muscle combined muscle index (AM-MI)), as well as pre-treatment BMI. The index with the highest area under the curve (AUC) was selected for use in determining a binary categorical variable for sarcopenic status. The cutoff used was determined by the locus with the highest combination of sensitivity and specificity. Sarcopenia was defined as having a “low” muscle index.

Outcomes

Overall survival and local progression were calculated from the end of radiation treatment to a censoring event or most recent follow-up. Local progression events included persistence of disease or recurrence within the treatment field on clinical exam, nasopharyngoscopy, or imaging. Persistent weight loss was defined as significant weight loss (>10% baseline) during treatment and failure to regain this weight six months after treatment. Toxicities were evaluated using Common Terminology Criteria for Adverse Events version 5. Toxicities occurring within three months of treatment were considered acute. Only severe toxicities, grade 3 and higher were included in this analysis. Treatment delays included any patient who missed two or more treatment days (excluding holidays and weekends).

All outcomes were stratified by possible confounders, including T stage, N stage, American Joint Committee on Cancer (AJCC) 8th edition staging, HIV status, gender, disease site, and presence of feeding tube prior to radiation.

Statistical analysis

Significance was set at p=0.05. All statistics were performed on SPSS v23.0.0.2 64-bit edition (IBM Inc., Armonk, NY). Pearson Chi-square tests of independence were performed for patient and treatment characteristics, with respect to sarcopenic status. The Bonferroni method was applied when multiple variables were evaluated simultaneously. Cutoffs for continuous variables were performed with receiver operating characteristic curves. Time-to-event was evaluated using the Kaplan-Meier method. Toxicities were evaluated using binomial logistic regression.

## Results

Sarcopenic status

**Figure 2:**
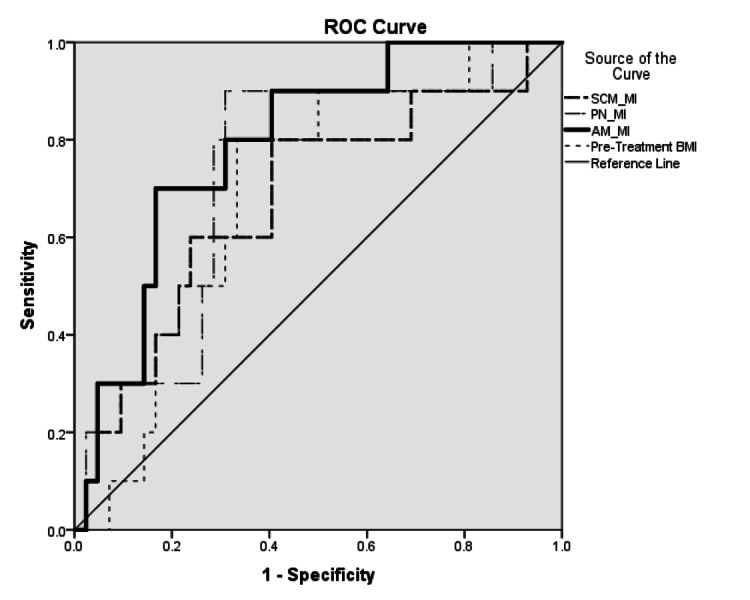
Receiver operating characteristic curve contrasting the sternocleidomastoids, the posterior neck muscles, all neck muscles combined, and pre-treatment body mass index AM-MI: sternocleidomastoid and posterior neck muscle combined muscle index, BMI: body mass index, PN-MI: posterior neck muscle index, ROC: receiver operating characteristic, SCM-MI: sternocleidomastoid muscle index

The highest AUC of 0.790 belonged to AM-MI (p=0.005). The only other statistically significant index was PN-MI (AUC=0.736, p=0.022). Both SCM-MI (AUC=0.679, p=0.082) and pre-treatment BMI (AUC=0.695, p=0.057) performed worse than PN-MI (Figure 2). A cutoff of 9.3 mm2/m2 was selected due to the highest combination of sensitivity (80.0%) and specificity (69.0%). Patients with a PN-MI lower than the cutoff were categorized as sarcopenic.

Patient and treatment characteristics

As expected, sarcopenia correlated with lower BMI (p=0.007) and oral cavity location (p=0.006). However, no statistically significant differences were identified in other baseline characteristics, such as gender, HIV status, presence of feeding tube prior to radiation or TNM staging (Table 1).

**Table 1:** Baseline characteristics AJCC: American Joint Committee on Cancer, HIV: human immunodeficiency virus, PEG: percutaneous endoscopic gastrostomy, NS: not significant

		All muscle-muscle index				p-value
		Sarcopenic		Non-sarcopenic		
		n (%)		n (%)		
Age (median)		59		58		NS
Gender	Male	17 (85)		25 (80.6)		0.69
	Female	3 (15)		6 (19.4)		
Pre-treatment BMI (median)		23.53		27.32		0.007
Anatomical site	Oral cavity	8 (38.1)		2 (6.5)		0.006
	Oropharynx	4 (19.0)		18 (58.1)		
	Hypopharynx‎/larynx	8 (38.1)		11 (35.5)		
	Salivary gland	1 (4.8)		0		
T stage	T1	3 (14.3)		4 (12.9)		0.592
	T2	5 (23.8)		13 (41.9)		
	T3	8 (38.1)		9 (29.0)		
	T4	5 (23.8)		5 (16.1)		
N stage	N0	5 (23.8)		9 (29.0)		0.592
	N1	4 (19.0)		7 (22.6)		
	N2	11 (52.4)		11 (35.5)		
	N3	1 (4.8)		4 (12.9)		
AJCC 8th staging	Stage I	1 (4.8)		4 (12.9)		0.219
	Stage II	2 (9.5)		8 (25.8)		
	Stage III	6 (28.6)		9 (29.0)		
	Stage IV-A/IV-B	12 (57.1)		10 (32.3)		
PEG present before radiation	No	14 (66.7)		25 (80.6)		0.253
	Yes	7 (33.3)		6 (19.4)		
HIV status	HIV negative or unknown	18 (90.0)		29 (93.5)		0.645
	HIV positive	2 (10.0)		2 (6.5)		

Treatment characteristics were similar across both groups (Table 2). Concurrent chemoradiation was administered in 83.9% of our patients, with weekly cisplatin given in all but two cases, where carboplatin and paclitaxel were used in one and cetuximab used in the other. The median follow-up for the entire cohort was 30.22 months.

**Table 2:** Treatment characteristics Chemo: chemotherapy, Gy: Gray, PEG: percutaneous endoscopic gastrostomy, post-op: post-operative, NS: not significant

		All muscle-muscle index				p-value
		Sarcopenic		Non-sarcopenic		
		n (%)		n (%)		
Radiation dose (Gy, median)		70		70		NS
Radiation fractions (median)		35		35		NS
Total elapsed radiation days (median)		47		49		NS
Days delayed (median)		2		2		NS
Concurrent chemo	No	3 (15.8)		4 (12.9)		0.775
	Yes	16 (84.2)		27 (87.1)		
PEG tube placed during treatment	No	11 (73.3)		20 (80.0)		0.625
	Yes	4 (26.7)		5 (20.0)		
Persistent weight loss	No	17 (81.0)		29 (93.5)		0.163
	Yes	4 (19.0)		2 (6.5)		
Post-op status	No prior surgery	10 (47.6)		22 (71.0)		0.089
	Post-op	11 (52.4)		9 (29.0)		

Outcomes

**Figure 3:**
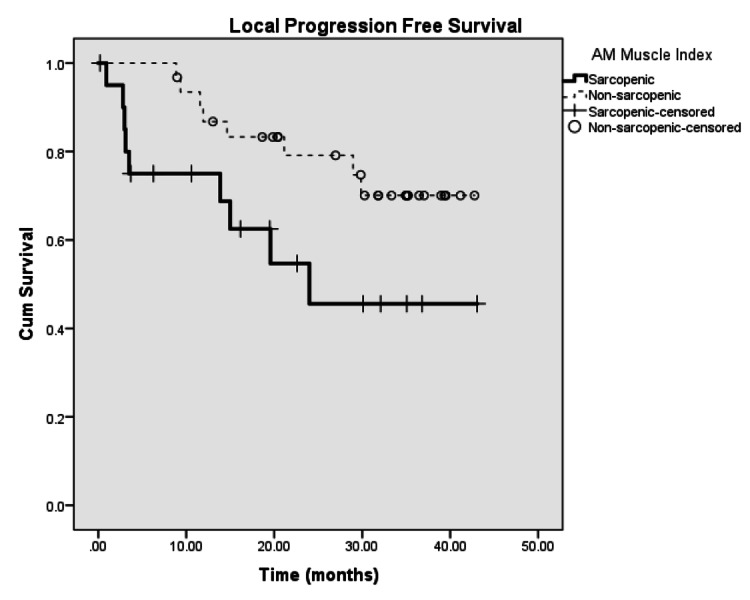
Kaplan-Meier curve for local progression-free survival between sarcopenic and non-sarcopenic patients AM: sternocleidomastoid and posterior neck muscle combined

Median local progression-free survival in sarcopenic patients was 25.81 months, while those without sarcopenia was 35.40 months (p=0.026, Figure 3). On univariate Cox regression, sarcopenia (HR=4.314, p=0.003) and percutaneous endoscopic gastrostomy (PEG) tube placed prior to treatment (HR=2.78, p=0.039) were associated with local progression. On multivariate analysis, only sarcopenia was significant for local progression-free survival (LPFS) (p<0.001).

**Figure 4:**
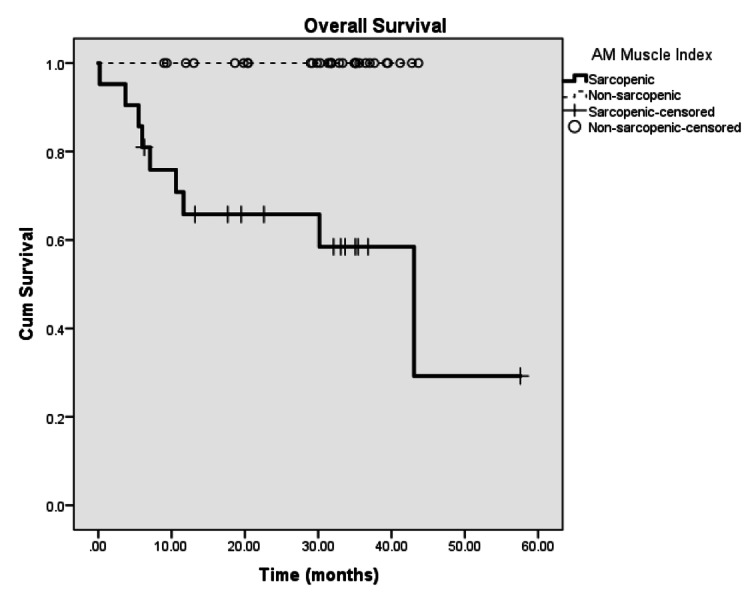
Kaplan-Meier curve for overall survival between sarcopenic and non-sarcopenic patients AM: Sternocleidomastoid and posterior neck muscle combined

Median survival in sarcopenic patients was 34.41 months, while their counterparts with high AM-MI was 42.30 months (p<0.001, Fig. 4). However, no censoring events occurred in the non-sarcopenic cohort (despite n=31), and Cox regression was not performed.

Acute grade 3+ toxicities were significantly more common in the sarcopenic group (HR 5.71, p=0.008). In the sarcopenic group, 10/21 (47.6%) experienced grade 3 or higher toxicities, compared to 6/31 (19.4%) patients in the non-sarcopenic group. In addition, persistent weight loss (p=0.181) or prolonged treatment delays (p=0.625) were not associated with sarcopenic patients.

## Discussion

The psoas muscles’ dominance as a proxy for sarcopenia/cachexia and predictive ability for outcomes in GI cancers led to a search for alternatives in H&NCP with similar traits. The ideal candidate needed to be within the H&N region, largely independent of patient positioning and resistant to disuse atrophy. The posterior neck muscles and sternocleidomastoids fit all requirements except for the centrally located transversospinales, which can vary significantly based on patient positioning. Ufuk et al. reported correlations between the sternocleidomastoids and paravertebral muscles with the psoas muscles [23]. Therefore, our study aimed to evaluate clinical correlates using the same muscle groups.

To our knowledge, this is the first study contrasting the clinically identified component muscle groups of the H&N in relation to radiotherapy treatment outcomes in sarcopenic H&NCP. Our results suggest that the combination of the SCMs and paravertebral muscles (AM-MI) identified sarcopenia more robustly than either muscle group alone or pre-treatment BMI. Moreover, all proxies of radiographic sarcopenia (SCM-MI, PN-MI, AM-MI) were better at predicting local progression. In our study, sarcopenic patients were more likely to locally progress and experience severe toxicities. For survival analysis, we saw a qualitative decrease in sarcopenic patients, but hesitate to draw statistical conclusions without a censoring event in the non-sarcopenic arm. Our initial working hypothesis was that sarcopenic patients are unable to tolerate treatment as well as non-sarcopenic patients. Then, increased rates of severe toxicities should lead to treatment delays, undertreated cancers from accelerated repopulation, higher rates of local progression, and ultimately, worse survival. However, both cohorts were similar in total treatment time; yet sarcopenic patients still progressed quicker. This suggests these patients either have a more biologically aggressive cancer, or there may be a metabolic threshold associated with successful radiotherapy treatment. Additional confirmatory studies are needed on both fronts.

Other contemporary retrospective studies used the sternocleidomastoids to predict for sarcopenic outcomes. Ganju et al. published the largest series to date with 246 H&NCP [10]. Sarcopenia was associated with worse overall survival, progression-free survival, and radiation treatment breaks >1 week. Even in a high-risk sarcopenic population, Ganju et al. reported an unusually high (14%) rate of radiation breaks of one week or longer. Our baseline and treatment characteristics were fairly like the University of Kansas study, with the only differences found in the proportion of obese patients and racial demographics. Patients in our study were primarily Hispanic, non-obese males compared to the University of Kansas’ higher proportion of white, obese males. Sarcopenic obesity correlated with poor outcomes in the GI literature, but a Montefiore Medical Center published results detailing sarcopenic obesity as a protective factor for overall survival in H&NCP [24]. Unfortunately, the literature does a poor job of defining sarcopenia, which may explain the heterogeneous results. Defining the volume of interest has been approached by using automated contouring of an entire axial slice, parts of an axial slice, or focused muscle groups, while cutoffs have been defined using population-based, cohort-based, disease site-based, and outcome-based values. Further studies with clear definitions for sarcopenia are needed to clarify their relationship to treatment outcomes.

The generalizability of our results is limited by its treatment in a safety net hospital (SNH), additional confounders, retrospective nature, and sample size. NCDB analyses found that patients treated at SNHs were associated with more advanced stages, poorer surgical outcomes, and racial minority demographics [25,26]. Confounders for sarcopenia secondary to cancer cachexia include geriatric sarcopenia [3], end-stage chronic obstructive pulmonary disease (COPD)-related cachexia, AIDS wasting syndrome, and poor mechanical intake, all of which are radiographically and clinically indistinguishable. Karsten et al. identified sarcopenia as a predictor for prolonged feeding tube dependency and is the only study thus far in H&NCP [27]. Our results suggest that feeding tube insertion during radiation or before was not associated with sarcopenia. This may be due to variations with individual, institutional approaches towards multidisciplinary care. Forty-five percent of our sarcopenic patients were identified for high nutritional risk and underwent feeding tube placement; however, 75% of these patients had their feeding tubes placed even prior to ever setting foot in the radiation oncology department. Additionally, the highest risk patients for geriatric sarcopenia are over the age of 70, few of which fell in our study (n=6). For AIDS wasting syndrome, we are limited by the known HIV status of our patients since it is not a part of oncologic staging; however, out of the four patients with known HIV+, all were taking their anti-retroviral medications and had never experienced an AIDS-defining illness. Moreover, most patients received concurrent chemotherapy with cisplatin monitored by weekly complete blood counts (CBCs). In our patient population, 96% were heavy smokers, with many comorbid for COPD. In summary, we acknowledge that confounders for cancer cachexia exist, but we are limited by the retrospective nature of our study to truly elucidate these differences. Despite our small sample size, the data generated were able to convey a strong signal with poor outcomes. All previously mentioned confounders were evaluated for effect modification via stratified Kaplan-Meier analysis, and no significant effect modifiers were found. Specifically for the oral cavity subsite, there was a correlation with sarcopenia but no effect modification on outcomes.

The literature suggests that sarcopenia secondary to cancer cachexia is highly prevalent in H&NPC. Identifying a simple radiographic biomarker that can risk-stratify high-risk patients in need of interventional nutrition could improve oncologic outcomes. In addition to validation of these radiographic biomarkers in prospective studies, further studies should involve direct interventions to the cachectic process. Anti-inflammatory medications, anabolic/catabolic medications, nutritional/functional interventions are the four areas of ongoing research currently. An ongoing phase III trial in the UK called MENAC (Multimodal-Exercise, Nutrition and Anti-inflammatory medication for Cachexia) aims to utilize a holistic approach to an exceedingly complex problem [28].

## Conclusions

In locally advanced H&NCP undergoing radiotherapy at a safety net hospital, a single axial slice of the C3 paravertebral muscles or sternocleidomastoids can be used to identify sarcopenia. Sarcopenia was independently associated with worse local progression-free survival and severe toxicities. Additional prospective studies are needed to confirm the biology of tumors in sarcopenic patients, as well as the metabolic relationship to treatment outcomes.
